# GTP-Bound Escherichia coli FtsZ Filaments Are Composed of Tense Monomers: a Dynamic Nuclear Polarization-Nuclear Magnetic Resonance Study Using Interface Detection

**DOI:** 10.1128/mbio.02358-22

**Published:** 2022-10-10

**Authors:** Kelsey M. McCoy, Keith J. Fritzsching, Ann E. McDermott

**Affiliations:** a Department of Chemistry, Columbia University, New York, New York, USA; KUMC

**Keywords:** FtsZ, cytoskeleton, nuclear magnetic resonance, protein-protein interactions, structural biology

## Abstract

FtsZ filaments are the major structural component of the bacterial Z ring and are drivers of bacterial division. Crystal structures for FtsZ from some Gram-positive bacteria in the presence of GTP analogs suggest the possibility of a high-energy, “tense” conformation. It remains important to elucidate whether this tense form is the dominant form in filaments. Using dynamic nuclear polarization (DNP) solid-state nuclear magnetic resonance (NMR) and differential isotopic labeling, we directly detected residues located at the intermonomer interface of GTP-bound wild-type (WT) Escherichia coli FtsZ filaments. We combined chemical shift prediction, homology modeling, and heteronuclear dipolar recoupling techniques to characterize the E. coli FtsZ filament interface and demonstrated that the monomers in active filaments assume a tense conformation.

## INTRODUCTION

FtsZ is a highly conserved cytoskeletal protein present in nearly all known bacteria and archaea (1–10). It is the major structural component of the Z ring, the first structure assembled during bacterial cytokinesis (11). FtsZ is present in the cytoplasm as a mix of soluble monomers, dimers, and transient oligomers (12–14), which assemble into dynamic filaments (15–17) that treadmill around the site of division—i.e., the midcell in fission bacteria such as Escherichia coli (18–21)—serving as a scaffold for downstream divisome factors (22, 23) and playing a role in membrane constriction (24–26).

*In vitro*, FtsZ reversibly assembles in the presence of MgCl_2_, KCl, and GTP (13, 27–29). FtsZ filaments assemble cooperatively (18, 30) with a GTP bound at each interface (13, 31); the GTPase active site is split such that individual monomers can bind, but not hydrolyze, GTP. Electron microscopy (EM) and kinetic data have led to a model where GTP binding precipitates monomer assembly into primarily straight filaments (9, 29, 32, 33). Over time, the filaments progressively curve and eventually disassemble, in correlation with GTP hydrolysis (32, 33). Because FtsZ assembles into single protofilaments composed of homomonomers, these conformational changes must be due to a corresponding conformational change within the monomers. However, until recently, only a single monomer form was observed by means of X-ray crystallography.

**FIG 1:**
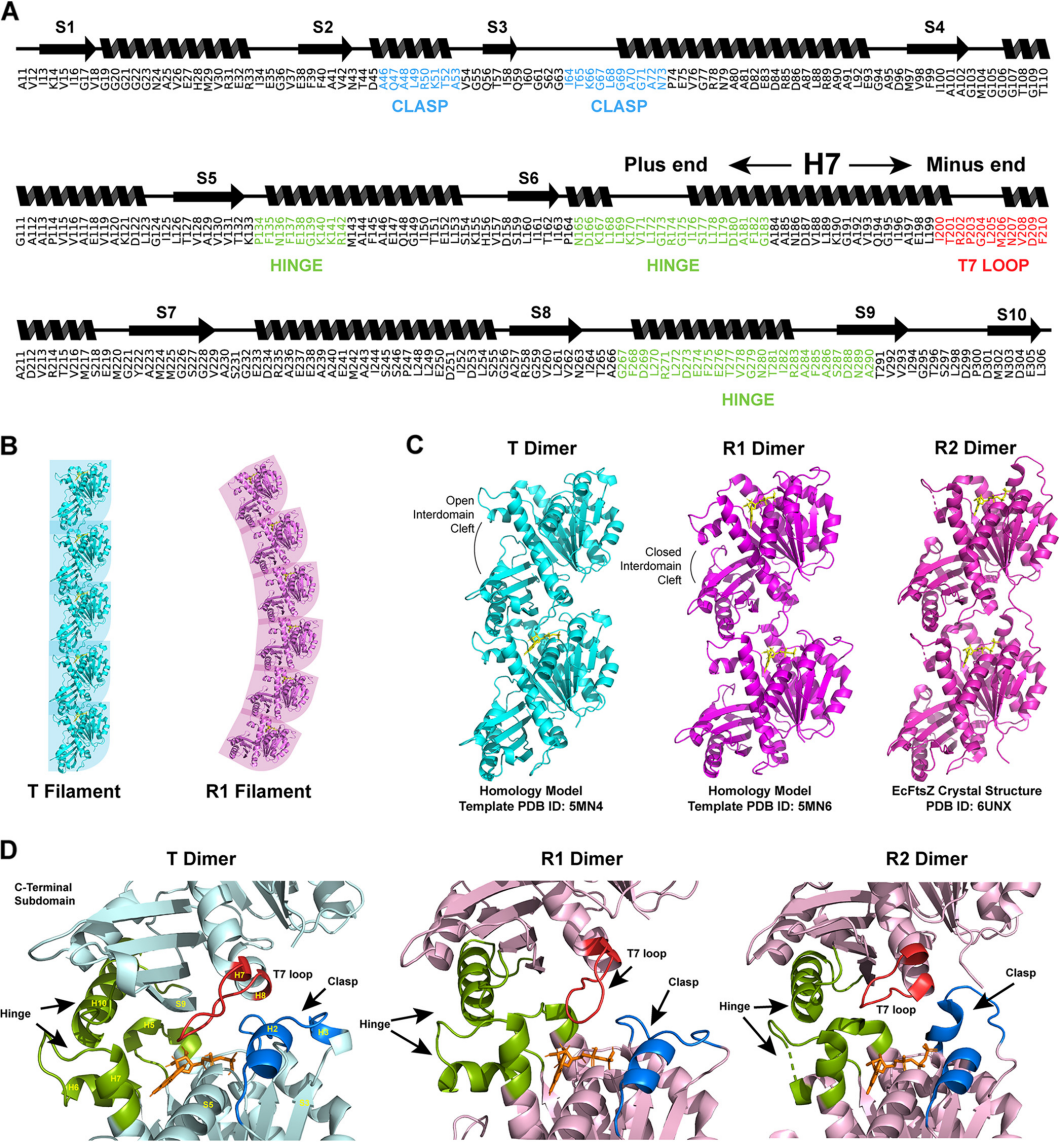
(A) Wire diagram and primary sequence of the structured domain of E. coli FtsZ used in this study with the interface regions labeled. (B) Structural representation of the filament structures of straight and curved filaments as composed of tense and relaxed monomers, respectively. PDB IDs 5MN4 (tense monomer) and 5MN6 (relaxed monomer) (41). (C) Structures of the three E. coli dimer models used in this study. Models T and R1 were constructed by templating the E. coli FtsZ sequence against dimers inferred from the crystal structures of S. aureus FtsZ monomers (PDB IDs 5MN4 [T] and 5MN6 [R1]). The R2 model was constructed directly from the E. coli FtsZ crystal structure (PDB ID 6UNX [73]).

In Staphylococcus aureus FtsZ (SaFtsZ), a second monomer conformation has been observed when FtsZ is bound to a small-molecule inhibitor, PC190273 (34, 35), after certain mutations (18, 36) and when bound to GTP mimics (37, 38). PC190273 binding to Bacillus subtilis FtsZ filaments suggests that the interdomain cleft is open, consistent with the tense (T) state (39). Additionally, the T state roughly aligns with an ~6-Å cryo-EM density map of E. coli FtsZ (EcFtsZ) filaments (18) and maps well to a straight filament (Fig. 1B). The current model posits that FtsZ monomers preferentially adopt a relaxed (R) state (the canonical FtsZ structure) in the cytoplasmic pool and switch into the T state upon nucleation and polymerization. As the filaments hydrolyze GTP, the monomers revert to the relaxed form and the filaments disassemble.

However, to date, a T state has been crystallized only from SaFtsZ, and the evidence that it is a conserved, active state is minimal. Notably, the inhibitor used to stabilize SaFtsZ does not induce polymerization in EcFtsZ (40), and two recently published EcFtsZ crystal structures were both in the R state (41, 42). The goal of this study was to determine whether the monomers within active, GTP-bound E. coli FtsZ filaments are in the T or R state. We use magic angle spinning (MAS) nuclear magnetic resonance (NMR) under cryogenic dynamic nuclear polarization (DNP) conditions (115 K) to directly observed GTP-bound, full-length EcFtsZ filaments at atomic resolution. By focusing on the monomer-monomer interface within the filament, we were able to distinguish between T state monomers and R state monomers and determine that GTP-bound EcFtsZ filaments are composed primarily of T state monomers.

MAS NMR is an important structural technique that is well suited for the study of protein oligomers. MAS NMR has contributed greatly to our understanding of the structure of amyloid fibrils (43), membrane proteins (44), and other difficult-to-crystallize, insoluble protein systems (45). Experiments such as REDOR (rotational-echo double resonance) (46, 47) and TEDOR (transferred echo double resonance) (48) use the heteronuclear dipolar coupling to measure internuclear distances with a high degree of precision (49, 50). DNP increases the viability of such experiments by drastically reducing the spectrometer time required (51, 52). DNP uses cryogenic temperatures, paramagnetic doping, and high-powered microwave radiation to enhance NMR signals on the order of 70 to 100× for typical large proteins (53–59).

While large proteins (>200 residues) remain difficult to study using traditional MAS NMR methods that rely on full spectral assignments, the availability of homology modeling, crystal structures, and modern spectroscopy techniques allows targeted biological questions to be asked. FtsZ is one such large protein. EcFtsZ has 383 total residues, consisting of a short disordered N-terminal tail (~residues 1–10), a structured domain (Met9-Gly316 [PDB ID 6UNX] [41] and Ala11-Gly316 [PDB ID 6LL6] [42] made up of two subdomains, and a long, disordered C-terminal tail. However, the proliferation of FtsZ crystal structures makes FtsZ a good target for exploring the applicability of MAS NMR and DNP to large, biologically interesting systems.

## RESULTS AND DISCUSSION

### Modeling the EcFtsZ interface.

Two FtsZ monomer conformations have been identified in S. aureus: a relaxed, low-energy conformation that corresponds to the canonical FtsZ monomer structure and a tense, high-energy conformation that has been posited to represent the active, polymerized form of the monomer (18, 34, 35). Compared to the R state, the T-state monomer’s C-terminal subdomain is rotated and moved away from the N-terminal subdomain by nearly 30° and is characterized by an open interdomain cleft (18). In the context of the filament, the T-state monomer is aligned in the crystal in a manner that appears to create a straight filament with the characteristic 44-nm monomer spacing seen in EM images (18) (Fig. 1). However, inhibitor binding, mutagenesis, or the use of GTP mimics must be used to trap the monomer in the T state for crystallization (18, 34, 37), and the T state has been crystallized only in S. aureus.

The active form of FtsZ is the filament. Thus, the active form of the monomer is the form that is present in the active (straight) filament. Assuming that the two major forms of the monomer are the T and R states, we set out to identify which state is present in E. coli FtsZ filaments. Because FtsZ forms single-stranded filaments composed of a single monomer, the monomer-monomer interface is unique to the filament and can, in principle, be used to characterize the active monomers. In order to understand the differences in the monomer-monomer interface between the T and R states, we constructed three models of the EcFtsZ dimer. A T-state dimer model was generated by defining a dimer based on the crystal contacts in the PDB file of the SaFtsZ T-state monomer (PDB ID 5MN4 [18]). This dimer was then templated against the EcFtsZ sequence and used to construct a homology model of the EcFtsZ dimer. This same method was used to construct an R-type dimer (model R1) (PDB ID 5MN6 [18]). Additionally, the recent EcFtsZ monomer structure (PDB ID 6UNX [41]) was used to define a second R-state dimer (model R2) based on the asymmetric unit’s crystal contacts (Fig. 1).

While all three models have similar interfaces, the T model interface contains nearly twice as many interface residues, defined as any residue with at least one carbon atom within 5 Å of the other monomer. Using this definition, the T model has 78 interface residues, the R1 model has 41 interface residues, and the R2 model has 38 interface residues. The majority of R1 and R2 interface residues are in what we term the hinge region of the interface (Fig. 1). The hinge region consists of the N-terminal tip of H7, the flexible loop between H7 and H6, and H10 on the opposing monomer (Fig. 1D). The hinge region is enriched in hydrophobic residues (particularly leucine and valine), and while the exact contacts differ between models, the hinge contains close contacts in all three models. The T model’s additional interface residues are present across all interface regions, including the T7 loop, the GTP-binding pocket, and what we call the clasp region. The clasp consists primarily of the S3 and H3 on the N-terminal side of the interface. In the T model, this region makes contacts with the T7 loop and H8 in the C-terminal subdomain. In the R1 model, the clasp region does not make contact with the neighboring monomer. In the EcFtsZ crystal structure reported by Schumacher et al. (41) (the R2 model), this loop extends across the interface and does make contact with part of the T7 loop; however, the contacts are substantially different than those in the T model (Fig. 1D).

### Characterizing the EcFtsZ interface.

**FIG 2:**
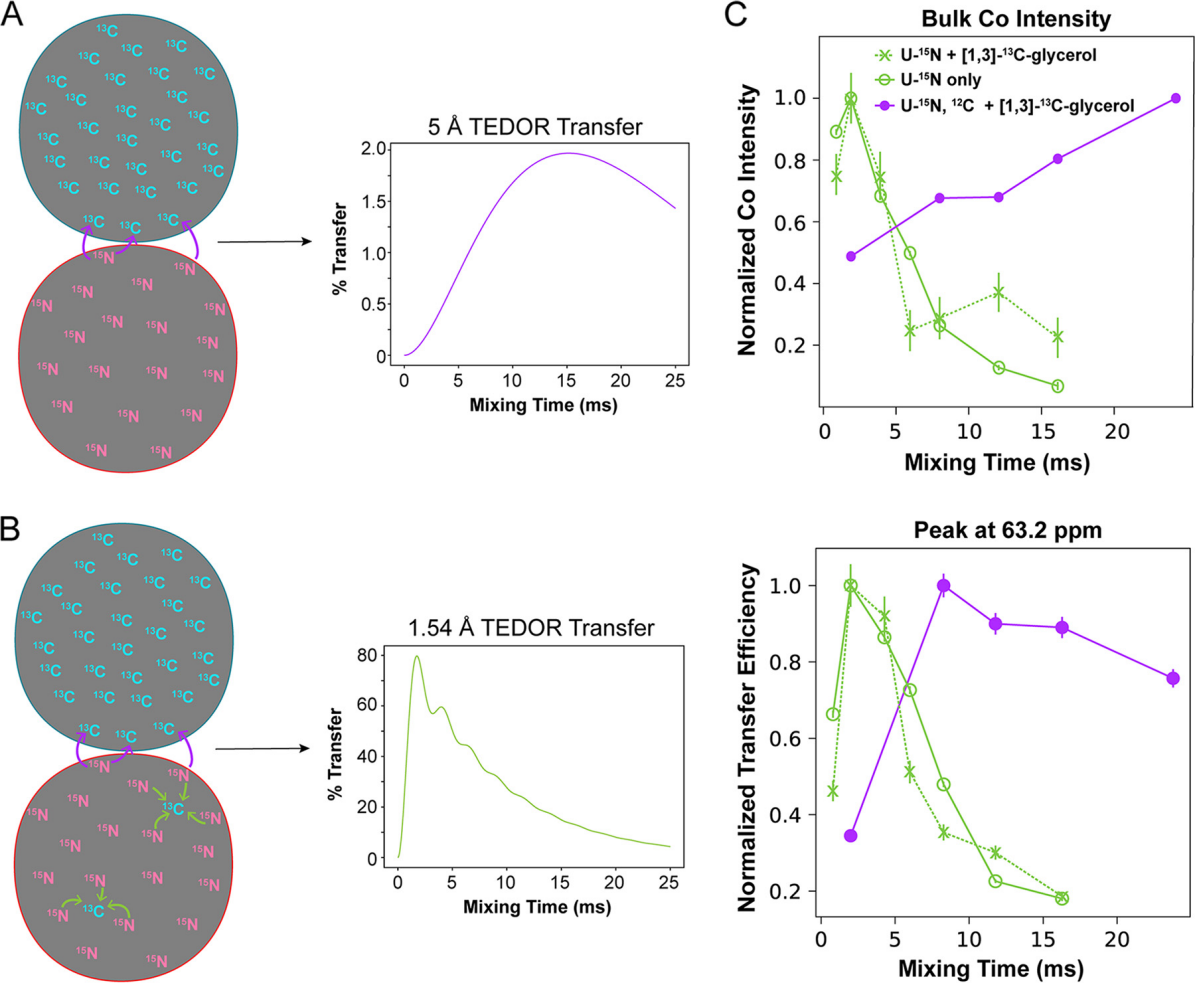
(A) Schematic of the differential isotopic labeling scheme used in this study. FtsZ monomers isotopically enriched only with ^15^N were mixed with monomers enriched only with ^13^C and polymerized together, forming a filament with randomly incorporated monomers. A spectroscopic technique (ZF-TEDOR) was employed to isolate only the ^13^C signals from atoms that are directly adjacent to ^15^N atoms. In the case of the differentially labeled filament, this should occur only at interfaces, leading to the simulated TEDOR buildup curve shown. (B) Schematic of differentially isotopically labeled dimers in the presence of a natural abundance of ^13^C in the ^15^N-labeled monomers. This changes the expected buildup curve, as shown in the simulated TEDOR curve. Both simulated TEDOR curves were calculated using an analytical expression that approximates TEDOR buildup (18). Parameters for the simulations were as follows: a single carbon either 1.54 Å or 5 Å from a single nitrogen (passive dipolar coupling of 0 Hz) with a T_2_ value of 8 ms, a scaler value of 1, and 10 Hz of second-order J coupling. (C) Experimental buildup curves from differentially labeled FtsZ monomers bound to GTP for both the bulk carbonyl intensity and an individual peak. FtsZ labeled with 1,3-^13^C-glycerol was mixed with U-^15^N-enriched FtsZ made with either natural-abundance glucose (purple) or ^12^C-glucose (99.9%) (green). FtsZ filaments composed of only U-^15^N enriched FtsZ made with either natural-abundance glucose were used as a control. The curves from the filaments made with natural-abundance glucose show a buildup pattern very similar to those from the U-^15^N-only filaments, with buildup peaking around 2.0 ms mixing, consistent with 1 or 2 bond distances. When filaments were made using ^12^C-glucose, the polarization transfer is consistent with long-distance, intermonomer contacts.

We used this information to design a set of solid-state MAS NMR experiments to probe the interface in active, GTP bound EcFtsZ filaments. Unlike X-ray crystallography, where filament structure must be inferred from crystal packing, MAS NMR allows us to study the filaments directly. However, MAS NMR is limited by low sensitivity. Long-range correlation experiments of the type used here are particularly inefficient, with as little as 2% transfer efficiency being common for 4- to 5-Å contacts (51) (Fig. 2A). We used dynamic nuclear polarization (DNP) to enhance our signal intensity an average of 70-fold. We were able to exploit DNP’s need for cryogenic sample temperatures (here averaging 115 K) to study full-length, wild-type (WT) EcFtsZ bound to GTP rather than a slowly hydrolyzing GTP mimic such as GMPCPP. By freezing our samples in liquid nitrogen within 10 min of GTP introduction, we trapped GTP-bound filaments in their active state for the duration of our experiments.

In order to isolate the longitudinal interface of EcFtsZ filaments, we employed a differential isotopic labeling scheme (Fig. 2) (51, 60). We prepared the ^15^N FtsZ samples with ^13^C-depleted glucose (99.9% ^12^C) to reduce contributions from intramonomer transfers due to background natural ^13^C abundance (Fig. 2). We prepared three samples of EcFtsZ filaments: 1,3-^13^C-glycerol + U-^15^N,^12^C mixed-label EcFtsZ (referred to below as the 1,3-glycerol sample) and 2-^13^C-glycerol + U-^15^N,^12^C mixed-label EcFtsZ (the 2-glycerol sample) were used to get broad coverage of interface residue atoms, and ^13^Cα-glycine- and ^13^Cβ-alanine-labeled EcFtsZ mixed with U-^15^N,^12^C EcFtsZ reduced spectral crowding (the ^13^Cα-Gly,^13^Cβ-Ala sample). Additionally, the 2-glycerol and ^13^Cα-Gly,^13^Cβ-Ala samples were fractionally deuterated on the ^13^C-enriched monomers in order to obtain suitable transverse (T_2_) relaxation values for the collection of long-mixing-time spectra. For each sample, we collected a series of one-dimensional (1D) z-filtered TEDOR (ZF-TEDOR) spectra at various mixing times and observed polarization buildup in a distance-dependent manner.

We identified 49 peaks in the 1,3-glycerol spectra, 83 peaks in the 2-glycerol spectra, and 94 peaks in the ^13^Cα-Gly,^13^Cβ-Ala spectra. It is noted that the while the peaks were confirmed in two data sets, the peaks were still picked judiciously by hand. Our analysis relies on the presence or absence of many such peaks, so small errors in this peak list would not affect the overall outcome of our analysis. ZF-TEDOR buildup curves were plotted for each peak by integrating a slice of the spectrum 1 ppm wide centered at the peak. Transfer efficiencies were calculated by dividing the integrated intensity of each peak by the integrated intensity of the same slice of the ^1^H-^13^C cross polarization spectrum (see Fig. S4 in the supplemental material). This procedure was repeated for the same regions of the U-^15^N sample spectra as a control. Figure 2C shows a representative buildup curve (Fig. S5). The transfer efficiencies have maxima at >12 ms, consistent with internuclear distances between 4 and 6 Å, indicating that the contacts observed are intermonomer.

10.1128/mbio.02358-22.4 FIG S4 ^13^C cross polarization (CP) spectra for DNP samples used in this study. The parameters for each spectrum are as follows: for 1,3-glycerol, ns = 32 scans; for 2-glycerol, ns = 16 scans; for ^13^Cβ-Ala,^13^Cα-Gly, ns = 64 scans; for U-^15^N, ns = 64 scans. All samples had an MAS frequency of 14,000 Hz. All spectra were processed with 50 kHz of exponential line broadening. Download FIG S4, JPG file, 0.2 MB. Copyright © 2022 McCoy et al. 2022 McCoy et al. https://creativecommons.org/licenses/by/4.0/ This content is distributed under the terms of the Creative Commons Attribution 4.0 International license.

10.1128/mbio.02358-22.5 FIG S5 ZF-TEDOR buildup curves for selected peaks. (A) Buildup curves from peaks in the 1,3-^13^C-glycerol + U-^15^N,^12^C sample spectra, 1,3-^13^C-glycerol + U-^15^N sample spectra, and U-^15^N EcFtsZ sample spectra. (B) Buildup curves from peaks in the 2-^13^C-glycerol sample spectra and the U-^15^N EcFtsZ sample spectra. Download FIG S5, JPG file, 0.3 MB. Copyright © 2022 McCoy et al. 2022 McCoy et al. https://creativecommons.org/licenses/by/4.0/ This content is distributed under the terms of the Creative Commons Attribution 4.0 International license.

Due to the lack of full resonance assignments for EcFtsZ, along with low signal-to-noise (S/N) in the ZF-TEDOR 1D spectra, full characterization of the EcFtsZ interface is not possible with the data set described here. However, using the dimer models described above, we generated predicted chemical shift lists to compare to the peaks present in the spectra. We predicted the chemical shifts of the three dimer models using SHIFTX2 (61) and extracted the chemical shifts of interface residues. In order to test the experimental data against the models, we identified predicted chemical shifts within ±0.5 ppm of each experimental peak and compared the predictions. A cutoff of ±0.5 ppm was used because it corresponds to the average accuracy of ^13^C chemical shift prediction using SHIFTX2 (61). Additionally, it corresponds to our estimated ^13^C line width.

We ranked each model’s prediction based on whether the predicted residue appeared at the interface, was expected to be labeled, or appeared on a lateral surface and could be explained by lateral contacts between bundled filaments. Most peaks were equally well explained by all three models, meaning that all three models had predicted chemical shifts within 0.5 ppm of the experimental peak that correspond to labeled atoms in interface residues. Predicted shifts from residues appearing at the interface and atoms that are expected to be labeled at >50% efficiency were ranked the highest, followed by labeled atoms appearing at a lateral surface, unlabeled atoms that do not appear at an interface, and, finally, peaks that cannot be explained by any predicted chemical shift.

Of the 219 total peaks identified across all three samples, the T model can account for all but 24 of them (Table 1). The R1 accounts for all but 58 peaks, and the R2 model fails to account for 75 peaks. Five additional peaks in the 2-glycerol spectra cannot be explained by any model used in the analysis. These could result from side chain conformations not seen in the crystal structures used to construct the models.

**TABLE 1:** Model comparison by sample

Sample	No. of exptl peaks			
	Total	Not explained by:		
		T model	R1 model	R2 model
1,3-Glycerol	50	3	14	17
2-Glycerol	75	10	16	22
^13^Cβ-Ala,^13^Cα-Gly	94	11	28	36

We also identified 29 peaks that are accounted for by only the T model or only an R model (Table 2). In some cases, the experimental peak is within 0.5 ppm of two predicted chemical shifts, in which case the peak may correspond to overlapping peaks. We included peaks that may correspond to either a unique peak or two peaks in a single model, so long as they were not well explained by the other models. Of the 29 peaks identified, 18 (62%) are best explained only by the T model, 6 (21%) are best explained only by the R1 model, and none are best explained by the R2 model. However, 5 (17%) are best explained by either the R1 or the R2 model, but not the T model. Several of the predicted assignments correspond to atoms in the same residue. Thr296 can explain the peaks at 59.9 ppm in ^13^Cα-Gly,^13^Cβ-Ala spectra (Cα), 72.5 ppm in the 1,3-glycerol spectra (Cβ), and potentially the peak at 22.1 ppm in the 2-glycerol spectra (Cγ2). The recurrence of the same residues across all 3 samples demonstrates that the same interface form is present across samples and these data are highly reproducible.

**TABLE 2:** Model comparison by peak

Peak (ppm)	Predicted assignment	Model	Sample
14.9	Ile200 Cδ1	T	2-Glycerol
20.3	Thr201 Cγ2	R1	2-Glycerol
20.3	Val171 Cγ2	R1	^13^Cβ-Ala,^13^Cα-Gly
22.1	Thr201/Thr296 Cγ2	T	2-Glycerol
22.4	Ala284/Ala286 Cβ	R1	^13^Cβ-Ala,^13^Cα-Gly
26.0	Leu169 Cδ1	T	^13^Cβ-Ala,^13^Cα-Gly
26.4	Ile294 Cγ1	T	1,3-Glycerol
32.9	Val171/Val262 Cβ	R1	2-Glycerol
33.4	Lys51/Met206 Cβ	T	1,3-Glycerol
43.1	Leu168 Cα	T	2-Glycerol
48.2	Gly20 Cα	T	^13^Cβ-Ala,^13^Cα-Gly
53.7	Arg202 Cα	T	1,3-Glycerol
57.2	Ser287/Ser177 Cα	R1/R2	^13^Cβ-Ala,^13^Cα-Gly
57.6	Ser287/Ser177 Cα	R1/R2	^13^Cβ-Ala,^13^Cα-Gly
59.9	Thr296 Cα	T	^13^Cβ-Ala,^13^Cα-Gly
61.8	Ser177/Thr65 Cα	R1/R2	^13^Cβ-Ala,^13^Cα-Gly
63.2	Pro134 Cα/Ser177 Cβ	R1	1,3-Glycerol
63.3	Thr65 Cα	T	^13^Cβ-Ala,^13^Cα-Gly
63.5	Ile200 Cα	R1/R2	2-Glycerol
64.6	Thr201 Cα	T	^13^Cβ-Ala,^13^Cα-Gly
66.4	Thr215 Cα	T	^13^Cβ-Ala,^13^Cα-Gly
67.5	Thr281 Cα	T	^13^Cβ-Ala,^13^Cα-Gly
72.5	Thr296 Cβ	T	1,3-Glycerol
158.8	Arg202 Cz	T	2-Glycerol
160.8	Arg174/Arg271 Cz	R1	2-Glycerol
181.5	Glu250 Cδ	T	1,3-Glycerol

The fact that the T model better accounts for the experimental data is evidence that the EcFtsZ filaments are primarily composed of T monomers. That some peaks are not well explained by the T model used in this analysis suggests that either some percentage of interfaces present in the sample contain R-state monomers—due either to filaments with mixed states or to nonpolymerized oligomers—or that the physiological interface differ from the models used for this analysis. With continued refinement of the model, and subsequent rounds of chemical shift prediction and analysis, we expect that more peaks will be well explained by the model.

**FIG 3:**
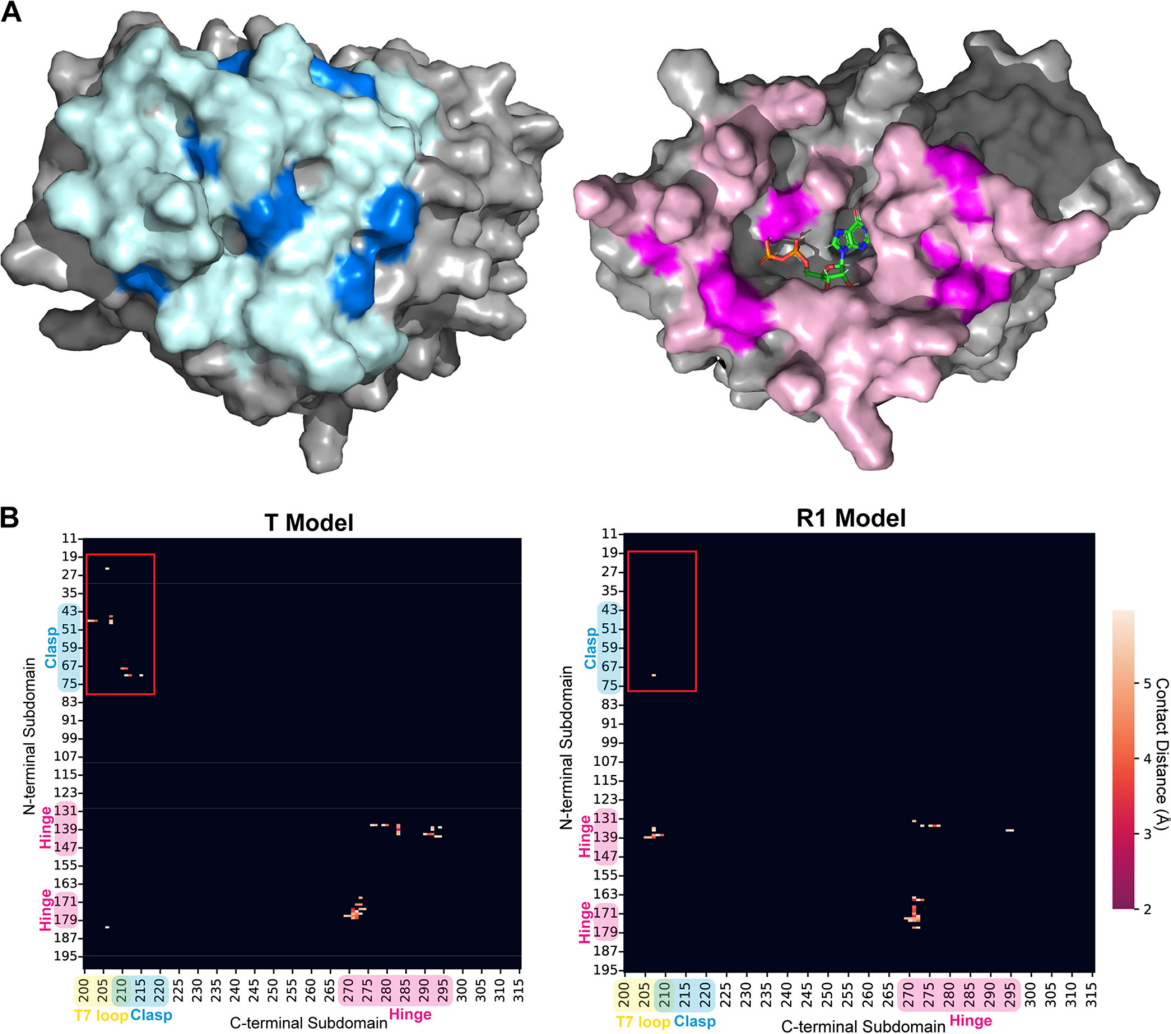
(A) EcFtsZ tense model dimer structure generated using homology modeling in Schrödinger Maestro templated against PDB ID 5MN4 (18). The locations of the predicted interresidue contacts are shown in light pink and light blue, and the locations of observed contacts are shown in dark pink and dark blue. (B) Contact map of the predicted T and R1 dimer interfaces showing sub-6-Å contacts between heavy atoms. Clasp region contacts are highlighted (red box), showing the presence of such contacts in the T model and absence in the R1 model.

Next, we took the identified residues and identified their location in the interface (Table 3). Glu250 and Thr281 are both located on the lateral surface of the monomer and likely represent lateral contacts between bundled filaments. Gly20 is located in the H1 helix and is part of the GTP-binding pocket. Leu168, Leu169, and Val171 are in the loop region between H6 and H7 and form the core of the hinge region on the N-terminal side of the interface. Ile294 and Thr296 are also part of the hinge region on the C-terminal subdomain. They are both located in the S9 strand, which faces H6 and the helical loop between S5 and H5 (Fig. 3).

**TABLE 3:** Interface residue locations

Residue	Model	Region
Gly20	T	GTP-binding pocket
Thr65	T	Clasp
Leu168	T	Hinge
Leu169	T	Hinge
Val171	R1	Hinge
Ile200	R1/T	T7 loop
Thr201	R1/T	T7 loop
Arg202	T	T7 loop
Thr215	T	Clasp
Glu250	T	Lateral contact
Thr281^*a*^	T	Lateral contact
Ile294	T	Hinge
Thr296	T	Hinge

a This peak may also be explained by Thr277.

**FIG 4:**
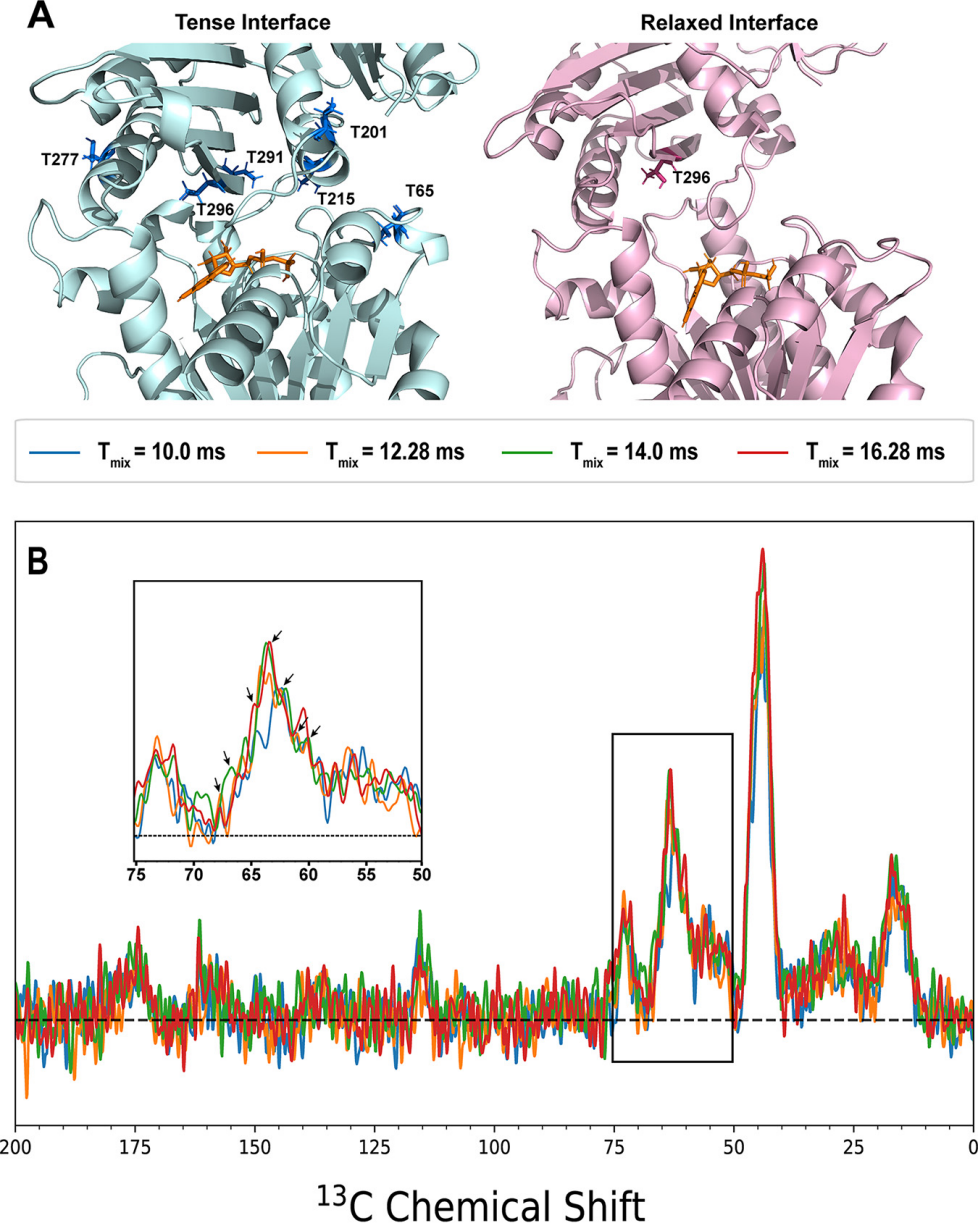
(A) Location of threonine residues at the interface in the T and R1 interface models. In the T model, six threonine residues are located within 5 Å of the interface. However, in the R1 model, structural rearrangement of the C-terminal subdomain moves all but one threonine (Thr296) away from the interface. (B) ^13^Cβ-Ala,^13^Cα-Gly ZF-TEDOR spectra for mixing times of 10.0 ms (blue), 12.28 ms (orange), 14.0 ms (green), and 16.28 ms (red) showing significant buildup between 60 and 70 ppm. All spectra were collected with 5,120 scans and processed with 50 Hz of exponential line broadening. (Inset) Closeup of the region between 60 and 75 ppm, with arrows indicating the 7 identified unique peaks.

The clasp region and the T7 loop are also present in the data. Ile200, Thr201, and Arg202 are in the T7 loop. Ile200 and Thr201 are expected to have contacts in the R models, but Arg202 is not, because the T7 loop is shifted away from the clasp in the R conformations. Thr65 is located in the clasp region on the N-terminal side of the interface and is predicted to have an interface contact in the R2 model and the T model. However, the chemical shift is significantly different between the two models. In the R2 model, Thr65 Cα is predicted to have a chemical shift of 61.9 ppm, whereas in the T model, it is predicted to be at 66.1 ppm, which aligns with the observed shift of 66.4 ppm. Thr215 is present in the C-terminal subdomain in the H8 helix (Fig. 1 and 4). In the R models, H8 is shifted up and away from the clasp region and Thr215 does not make contacts. Its presence in the data is a good indicator that the T state interface was being observed.

### Interface threonine shifts indicate a T monomer state.

In our models, threonines are good indicators of interface conformation. The T model interface contains six threonines (Thr65, Thr201, Thr215, Thr277, Thr291, and Thr296), whereas only one is present in either R model (Thr296 and Thr65) (Fig. 4A). In addition, threonine chemical shifts are easily distinguished from other residue types, making them a good marker for EcFtsZ interface conformation. While threonines can be difficult to specifically isotopically enrich due to isotope scrambling from the bacterial metabolism, we can exploit the same amino acid metabolic pathways and scrambling. For example, glycine Cα scrambles to serine and from serine to threonine (Fig. S6) Because of this, threonine Cαs were apparent in our ^13^Cα-Gly,^13^Cβ-Ala sample spectra (Fig. S4). While there is some overlap in the chemical shifts of serine Cαs with threonine Cαs, there are only 2 serines expected to be at the interface in any of the models (1 in the T model, 2 in the R1 model, and 1 in the R2 model), so serine Cα is not expected to contribute significantly to peaks between ~60 and 65 ppm.

10.1128/mbio.02358-22.6 FIG S6 (Top) 1D ^13^C DNP spectra of the ^13^Cβ-Ala^13^Cα-Gly EcFtsZ sample showing a cross polarization spectrum (blue, ns = 64) and a representative ZF-TEDOR spectrum (orange, 16.28-ms mixing time, ns = 5,120), annotated to show the isotopic scrambling pattern. (Bottom) ^13^Cβ-Ala,^13^Cα-Gly ^13^C CP spectrum (blue) and a natural-abundance ^13^C EcFtsZ CP DNP spectrum from the U-^15^N sample (orange). Download FIG S6, JPG file, 0.2 MB. Copyright © 2022 McCoy et al. 2022 McCoy et al. https://creativecommons.org/licenses/by/4.0/ This content is distributed under the terms of the Creative Commons Attribution 4.0 International license.

Figure 4B shows the long-mixing-time ZF-TEDOR spectra for the ^13^Cα-Gly,^13^Cβ-Ala sample. There is a large set of overlapping peaks evident between 60 and 70 ppm. Nine peaks were identified in this region, seven of which can be attributed to individual labeled atoms based on the chemical shift predictions for the T model (Table 4). Six of the seven peaks can be assigned to threonine Cαs, with the seventh peak corresponding to a serine Cα.

**TABLE 4:** Threonine peaks in ^13^Cβ-Ala,^13^Cα-Gly ZF-TEDOR spectra

Peak (ppm)	Predicted assignment
59.9	Thr296 Cα
60.9	Ser177 Cα
62.3	Thr291 Cα
63.3	Thr65 Cα
64.6	Thr201 Cα
66.4	Thr215 Cα
67.5	Thr281 Cα
68.9	Thr215/Thr277/Thr291 Cβ
69.7	Thr201 Cβ
70.6	Thr65 Cβ
71.7	Thr162/Thr291 Cβ
72.5	Thr296 Cβ
73.2	Glycerol
74.0	Glycerol

Five of the six predicted threonine Cα peaks can be seen in the spectra, with only Thr277 missing. Thr277 Cα is predicted to have a chemical shift of 66.96 ppm, which is 0.04 ppm away from the 0.5-ppm cutoff used in this analysis, so it is possible that the peak at 67.5 ppm is from Thr277 rather than Thr281. Regardless, the presence of five of the six predicted threonine Cα peaks in the ZF-TEDOR spectra indicates that the EcFtsZ filaments adopt a T monomer conformation similar to the T form of the SaFtsZ monomer.

Additionally, there is some signal in the region from 70 to 75 ppm in the ^13^Cα-Gly,^13^Cβ-Ala sample (Fig. 4B), which is centered around 73 ppm. While it is possible that this is from the glycerol peak expected to be at 74.9 ppm, it could also be due to an increased level of ^13^C at the threonine Cβ position over the 0.1% ^13^C background (Fig. S6). We identified 5 peaks between 68 and 73 ppm (68.9 ppm, 69.7 ppm, 70.6 ppm, 71.7 ppm, and 72.5 ppm). The peaks at 70.6 ppm and 72.5 ppm both appear in other data sets and can be assigned to Thr65 Cβ and Thr296 Cβ, respectively. The peak at 69.7 ppm can be assigned to Thr201 Cβ, and the peak at 68.9 ppm is within ±0.5 ppm of the predicted chemical shifts of Thr215, Thr277, and Thr291 Cβ. The 71.7-ppm peak is not within 0.5 ppm of any predicted interface threonine shifts and is likely due to a shift that is not present in the models.

### ZF-TEDOR buildup curve fitting.

In order to extract the internuclear distances from the ZF-TEDOR buildup curves, we implemented numerical simulations using SPINEVOLUTION (62). For the purpose of this analysis, we selected peaks corresponding to a single predicted chemical shift from the T model. We did not include peaks from the 1,3-glycerol spectra, however, because that data set contained fewer time points. Using this method, we identified 7 peaks in the 2-glycerol 1D ZF-TEDOR spectra and 12 peaks in the ^13^Cα-Gly,^13^Cβ-Ala sample spectra (Table S1).

10.1128/mbio.02358-22.7 TABLE S1 Unique peaks identified in the 2-glycerol and ^13^Cβ-Ala,^13^Cα-Gly spectra and their predicted assignments. Download Table S1, JPG file, 0.2 MB. Copyright © 2022 McCoy et al. 2022 McCoy et al. https://creativecommons.org/licenses/by/4.0/ This content is distributed under the terms of the Creative Commons Attribution 4.0 International license.

**FIG 5:**
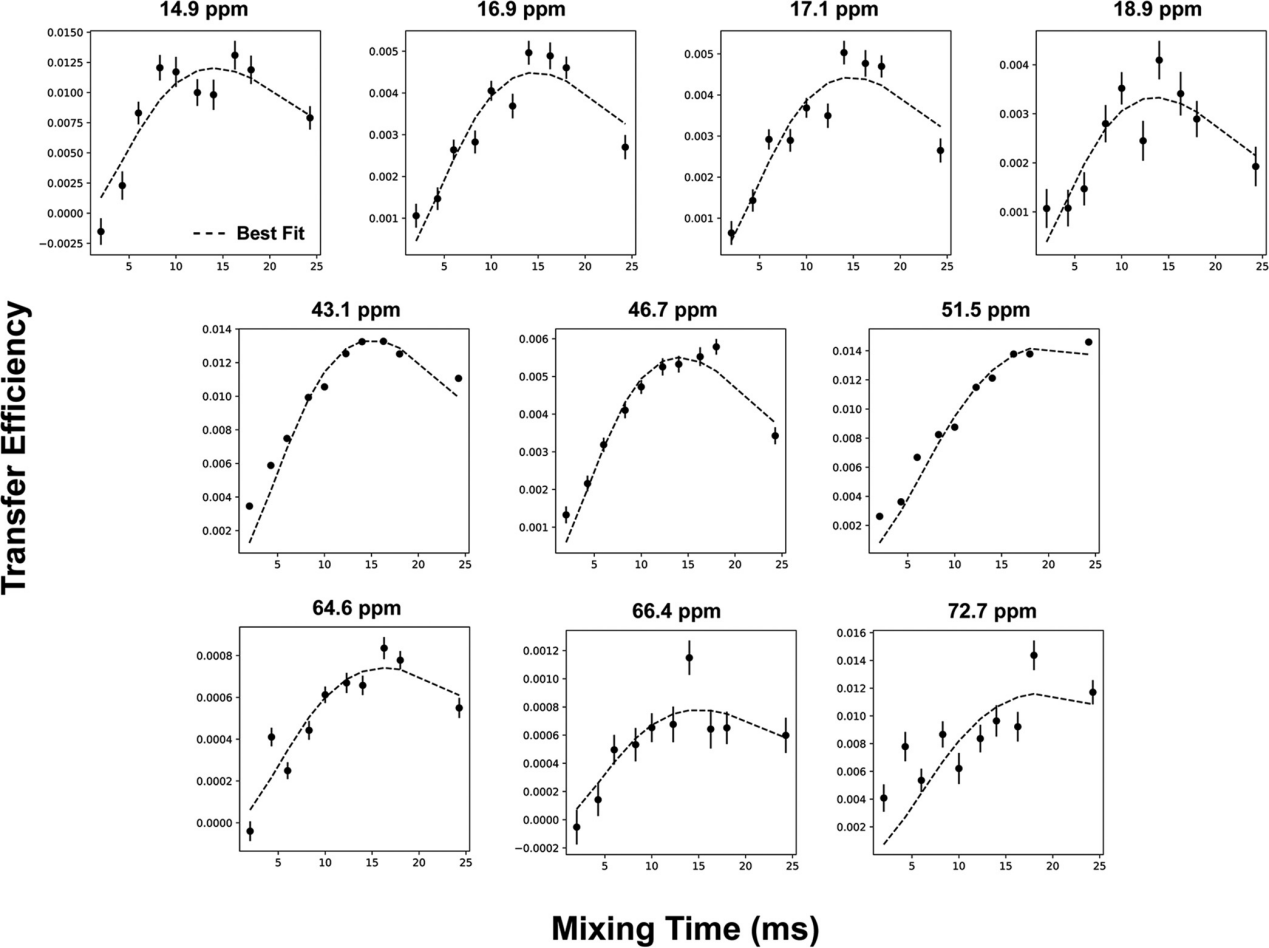
Best-fit curves for ZF-TEDOR buildup curves for unique peaks in 2-glycerol and ^13^Cβ-Ala,^13^Cα-Gly sample spectra. Fits were generated using SPINEVOLUTION and the Python lmfit library using an isolated C-N spin pair model. Fits had 10-Hz J coupling applied to simulate higher-order ^13^C-^13^C couplings present in the system.

This system gave good fits for 11 of the 19 buildup curves based on the root mean square error (RMSE) cutoff of 0.5 (Table 5; Fig. 5). We compared our measured distances to the predicted distances between each measured carbon and the nearest cross-interface nitrogen (Table 5). In most cases, the T model distance was closer to our measured distance than the distance in either R model. However, the model distances are generally longer than our measured distances, suggesting that the interface is tighter than it appears in crystal structures. In the cases of Ile176 Cγ2 and Ala72 Cα and Cβ, the measured distances agreed better with the R1 model than the T model predictions. The T1 model predicts a contact between Ile176 Cγ2 and a backbone nitrogen of Arg271 with a distance of 5.4 Å. In the R1 model, Arg271 is rotated so that its side chain nitrogens are facing Ile176, making a much shorter, 3.5-Å contact, in agreement with the measured distance of 3.5 ± 0.6 Å. In the T model, Ala72 makes contact with the backbone nitrogen of Thr215 of the opposing monomer, an ~7-Å distance. However, in both R models, Ala72 makes contacts with the backbone nitrogens in the T7 loop with distances between 5.4 and 7.8 Å. However, the agreement of the measured distances for T7 loop residues Ile200 and Thr201 suggests that the T7 loop is not in a conformation that can make close contacts with Ala72.

**TABLE 5:** Best-fit distances for unique peaks^*a*^

Peak (ppm)	Assignment	Distance (Å)				Region
		Best fit^*b*^	T model	R1 model	T2 model	
14.9	Ile200 Cδ1	4.9 ± 0.4	6.7	11.8	9.0	T7 loop
16.9	Ile200 Cγ2	5.0 ± 0.6	6.6	11.3	7.8	T7 loop
17.1	Ile176 Cγ2	3.5 ± 0.6	5.4	3.5	7.1	Hinge
18.9	Ala72 Cβ	5.0 ± 0.7	7.2	5.4	6.6	Clasp
43.1	Leu168 Cβ	4.07 ± 0.03	8.6	7.0	9.7	Hinge
46.7	Gly71 Cα	5.0 ± 0.3	4.1	7.9	9.1	Clasp
51.5	Ala72 Cα	5.2 ± 0.2	7.2	5.4	7.8	Clasp
64.6	Thr201 Cα	5.3 ± 0.2	4.7	11.7	11.5	T7 loop
66.4	Thr215 Cα	4.9 ± 0.6	6.9	13.5	6.8	Clasp
72.7	Thr296 Cβ	5.0 ± 0.7	8.1	7.5	11.5	Hinge

a All fitting parameters can be found in Table S2.

b Data are best fit distance ± jackknife fitting error.

10.1128/mbio.02358-22.8 TABLE S2 Best-fit parameters and the fit root mean square deviation (RMSD) for each of the 19 uniquely identified peaks. Download Table S2, JPG file, 0.3 MB. Copyright © 2022 McCoy et al. 2022 McCoy et al. https://creativecommons.org/licenses/by/4.0/ This content is distributed under the terms of the Creative Commons Attribution 4.0 International license.

**FIG 6:**
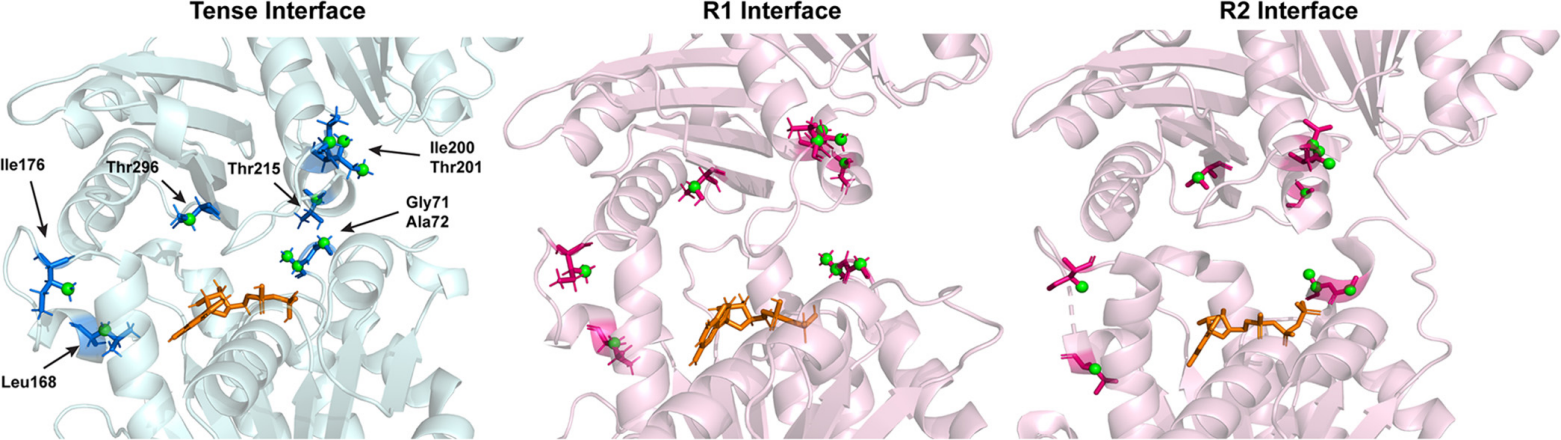
Residues with measured contacts in all three interface models, with selected residues labeled for comparison. Assigned atoms are shown in green (spheres). See Table 5 for measured and predicted contact distances for each model.

Both Ile176 and Ala72 are located in loops (Fig. 6) where we expect a degree of disagreement between our models and the protein structure. In the case of Ile176, it is possible that the side chain of Arg271 faces the opposing monomer as it does in the R1 model. Additionally, given that the hinge region is common to all three interfaces, Ile176 is not expected to discriminate well between models. Ala72, however, is in the clasp region, which we expect to be predictive of interface state. The agreement between our measured distances and the predicted distances of Ala72 contacts, however, must be read in the context of the other contacts in that region. Gly71 Cα is predicted to have a contact distance of 4.1 Å in the T model, which agrees better with our measured distance of 5.0 ± 0.3 Å than the R1-predicted distance of 7.9 Å. Thr215 is opposite Ala72 in the T1 model, and the measured distance of 4.9 ± 0.6 Å for Thr215 Cα agrees better with the T1 prediction of 6.9 Å than the R1 prediction of 13.5 Å.

Overall, the distances we were able to measure agree with a T-state interface. They also suggest that the interface is tighter than those observed in crystal structures. More work is needed to determine the precise contacts, which will allow more accurate mapping of the interface. Taken together, these data support a model of the EcFtsZ filament where the monomers are in the T conformation. The interface itself is tight, with contacts as short as ~4.0 Å, and the T7 loop extends into the GTP-binding pocket. The clasp region of the interface makes several close contacts across the interface, indicating that it is involved in stabilizing the filament in a straight conformation.

### Conclusions.

Using DNP ZF-TEDOR experiments, we were able to directly observe contacts between ^13^C and ^15^N nuclei at the EcFtsZ intermonomer interface. The lack of site-specific resonance assignments for the EcFtsZ monomer means that we cannot unambiguously assign peaks in the ZF-TEDOR spectra. However, the availability of crystal structures of FtsZ monomers allowed us to generate chemical shift predictions using homology models. Combining structural analysis of the models with chemical shift predictions allowed us to demonstrate that the T model best explains our data. In addition, the presence of multiple threonine resonances in our spectra indicates that the monomers are in a T state.

## MATERIALS AND METHODS

### Protein expression and purification.

EcFtsZ was expressed from a pET21b(+) plasmid generously provided by Anuradha Janakiraman at the City College of New York. All protein was expressed in BL21(DE3) chemically competent cells using 0.5 mM isopropyl-β-d-thiogalactopyranoside (IPTG). Isotopically enriched, protonated FtsZ was expressed using a 4:1 LB:M9 transfer with precursor concentrations as follows: for U-^15^N FtsZ, 1 g/L ^15^NH_4_Cl + 4 g/L d-glucose; for U-^15^N,U-^12^C FtsZ, 1 g/L ^15^NH_4_Cl + 2 g/L 99.9% ^12^C d-glucose; for the sparsely labeled ^13^C FtsZ, 1 g/L 1,3-^13^C-glycerol + 1 g/L NH_4_Cl + 1 g/L sodium bicarbonate (63). All isotopes were purchased from Cambridge Isotope Laboratory (Tewksbury, MA, USA). All biochemical reagents were purchased from Thermo Fisher Scientific (Waltham, MA, USA). GTP was purchased from Sigma Aldrich Millipore (St. Louis, MO, USA).

Partially deuterated EcFtsZ was expressed using M9 adaptation (64–66). Samples were prepared by growing 100% ^2^H_2_O LB-adapted BL21(DE3) cells transformed with pET21b(+):EcFtsZ in a 4-mL culture of ^2^H_2_O LB for 12 h. Five hundred milliliters of 100% ^2^H_2_O M9 medium was inoculated with 1% of the overnight cultures and grown to an optical density at 600 nm (OD_600_) of ~0.6, which took 18 to 20 h. Expression was induced with 0.5 mM IPTG in ^2^H_2_O and halted after 4 h. For the ^2^H_2_O, 2-^13^C-glycerol EcFtsZ, the M9 was prepared with 2 g/L 2-^13^C-glycerol, 1 g/L NH_4_Cl, and 2 g/L ^13^C-sodium bicarbonate. For the ^2^H_2_O, ^13^Cα-glycine, ^13^Cβ-alanine EcFtsZ, the M9 was prepared with 4 g/L ^12^C-glucose (99.9%), 1 g/L NH_4_Cl, 1 g/L ^13^Cα-glycine, 1 g/L ^13^Cβ-alanine, 0.1 g/L l-isoleucine, and 0.2 g/L α-ketoisovalerate.

EcFtsZ was purified using ammonium sulfate precipitation and calcium sedimentation, as described elsewhere (14, 67). Anion exchange chromatography was not deemed necessary based on comparative purity analysis (Fig. S1). Functional analysis was carried out using 90° light scattering (Fig. S2) and negative-staining EM (Fig. S3) (29).

10.1128/mbio.02358-22.1 FIG S1 SDS-PAGE gel stained with silver nitrate showing the relative purity of various purification methods. Lane 1, ladder; lane 2, calcium sedimentation followed by anion-exchange chromatography; lane 3, ammonium sulfate precipitation followed by calcium sedimentation; lane 4, ammonium sulfate precipitation followed by anion-exchange chromatography. Download FIG S1, JPG file, 0.2 MB. Copyright © 2022 McCoy et al. 2022 McCoy et al. https://creativecommons.org/licenses/by/4.0/ This content is distributed under the terms of the Creative Commons Attribution 4.0 International license.

10.1128/mbio.02358-22.2 FIG S2 Ninety-degree light scattering demonstrating that the purified EcFtsZ behaves as expected. EcFtsZ polymerization was induced by 1 mM GTP (blue); 1 mM GDP (red) does not induce any increase in scattering, demonstrating that GTP is necessary for polymerization. The buffer was 50 mM MES–NaOH (pH 6.5), 1 mM EGTA, 50 mM KCl, 10 mM MgCl_2._ All assays were performed at 30°C with stirring. Download FIG S2, JPG file, 0.1 MB. Copyright © 2022 McCoy et al. 2022 McCoy et al. https://creativecommons.org/licenses/by/4.0/ This content is distributed under the terms of the Creative Commons Attribution 4.0 International license.

10.1128/mbio.02358-22.3 FIG S3 Representative TEM images of EcFtsZ filaments under various buffer conditions. MMK2.5, 50 mM MES, pH 6.5-50 mM KCl-2.5 mM MgCl_2_; MM, 50 mM MES, pH 6.5-2.5 mM MgCl_2_; MEK, 50 mM MES, pH 6.5-50 mM KCl-2.5 mM EDTA; MMK2.5 + GTPγS, EcFtsZ polymerized in MMK2.5 buffer with 1 mM GTPγS instead of 1 mM GTP as a negative control. All buffers contained 50 mM MES–NaOH (pH 6.5), and all samples were polymerized with 1 mM GTP unless otherwise stated. Download FIG S3, JPG file, 0.5 MB. Copyright © 2022 McCoy et al. 2022 McCoy et al. https://creativecommons.org/licenses/by/4.0/ This content is distributed under the terms of the Creative Commons Attribution 4.0 International license.

### DNP.

All samples were polymerized in DNP buffer (50 mM MES [morpholineethanesulfonic acid]-NaOH [pH 6.5], 50 mM KCl, 20 mM MgCl_2_, 2 mM EDTA) diluted to 30% U-^12^C,U-^2^H glycerol, bringing the final buffer concentrations to approximately 5 mg FtsZ, 30 mM KCl, 12 mM MgCl_2_, 1.2 mM EDTA, 3 mM GTP, and 10 mM AMUPol (68) (H_2_O/D_2_O, ~65/35). Samples were centrifuged in Bruker 1.9-mm low-temperature rotors and promptly frozen in liquid nitrogen to preserve stable filaments.

DNP experiments were performed on a 600 MHz (14.1 T) Bruker Avance III-DNP system at the New York Structural Biology Center (NYSBC), which is equipped with a 395 GHz gyrotron and an HCN triple-channel probe. Additional DNP experiments were performed on a 600-MHz (14.1 T) Bruker Avance III-DNP system located at the Bruker Biospin Corporation in Billerica, MA, equipped with a 395 GHz gyrotron and an HCN triple-channel probe. All pulse sequences used in this study can be found at http://comdnmr.nysbc.org/comd-nmr-dissem/comd-nmr-solid.

DNP experiments were run using a spinning frequency (ω*_r_*/2π) of 14,000 ± 10 Hz and an estimated temperature of 115 ± 2.5 K. Standard π/2 pulse lengths of 1.5, 3.0, and 4.2 μs were used for the ^1^H, ^13^C, and ^15^N channels, respectively, corresponding to ω_1_/2π values of 169 kHz (^1^H), 83 kHz (^13^C), and 60 kHz (^15^N). Heteronuclear recoupling was done using ZF-TEDOR (69) with mixing times between 2.0 and 24.28 ms. ^1^H decoupling with an ω_1,_*_H_*/2π value of >70 kHz was performed using SPINAL-64 decoupling (70).

Experiments done at Bruker BioSpin (Billerica, MA) were collected with a spinning frequency, (ω*_r_*/2π) of 14,000 ± 10 Hz and an estimated temperature of 115 ± 2.5 K. Standard π/2 pulse lengths of 1.2, 3.0, and 4.15 μs were used for the ^1^H, ^13^C, and ^15^N channels, respectively, corresponding to ω_1_/2π values of 208 kHz (^1^H), 83 kHz (^13^C), and 60 kHz (^15^N). Heteronuclear recoupling was done using ZF-TEDOR using mixing times between 2.0 and 24.28 ms. ^1^H decoupling with an ω_1,_*_H_*/2π value of >80 kHz was performed using SPINAL-64 decoupling.

Spectra were processed using TopSpin (Bruker BioSpin). To account for low signal-to-noise ratios, peaks were picked by overlaying 1D ZF-TEDOR spectra and identifying peaks that occurred in at least 2 spectra. Data were analyzed in Python using the NMRglue library (71).

### Homology model construction.

EcFtsZ dimer models were constructed using the Schrödinger Maestro suite (Schrödinger release 2020-4: Schrödinger, LLC, New York, NY). A dimer was inferred from the Staphylococcus aureus crystal structure by choosing the symmetry mate that best resembled a filament for the tense (PDB ID 5MN4) and relaxed (PDB ID 5MN6) forms (18). The dimers were then templated against the wild-type EcFtsZ sequence to construct homology models. A second relaxed dimer model (R2) was constructed using the EcFtsZ crystal structure (PDB ID 6UNX) (41). The interface was inferred by choosing the symmetry mate that best resembled a filament. All chemical shifts were predicted using SHIFTX2 (61).

### Curve fitting.

In order to extract internuclear distances, the buildup curves of the identified peaks were fitted using numerical simulations generated by SPINEVOLUTION (62) as described in detail elsewhere (72). The RMSE was calculated for the fits along with RMSE/σ_data_ and $\bar{\text{data}}$—the coefficient of variation of the RMSE—as measures of goodness of fit. An acceptable fit is defined here as a fit with an RMSE/σ_data_ value of <0.5—i.e., the standard deviation of the residuals between the fit and the data (the RMSE) is less than half of the standard deviation of the data. If this value was over 0.5 across all fitting models, the curve was deemed too noisy for accurate fitting and was excluded. The fit error was estimated using jackknife error estimation.

### Data availability.

All spectra are available on the BMRB (ID 51610).
