# Supplementary figures and images for: GTP-Bound Escherichia coli FtsZ Filaments Are Composed of Tense Monomers: a Dynamic Nuclear Polarization-Nuclear Magnetic Resonance Study Using Interface Detection
Source: mBio. 2022 Oct 10;13(6):e02358-22. doi: 10.1128/mbio.02358-22 (PMC9765660; doi:10.1128/mbio.02358-22)

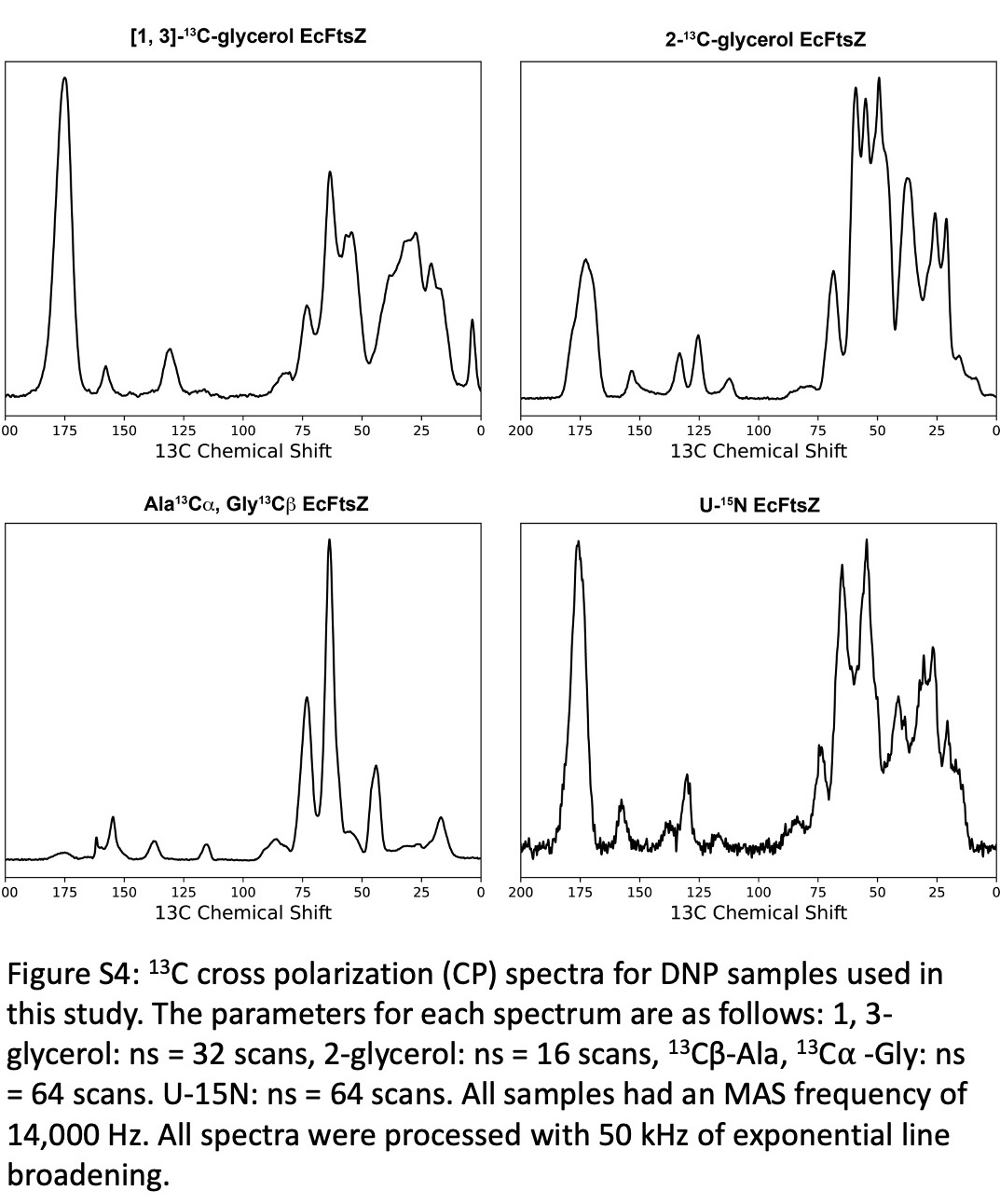

Supplement: FIG S4 [file mbio.02358-22-s0004.jpg]

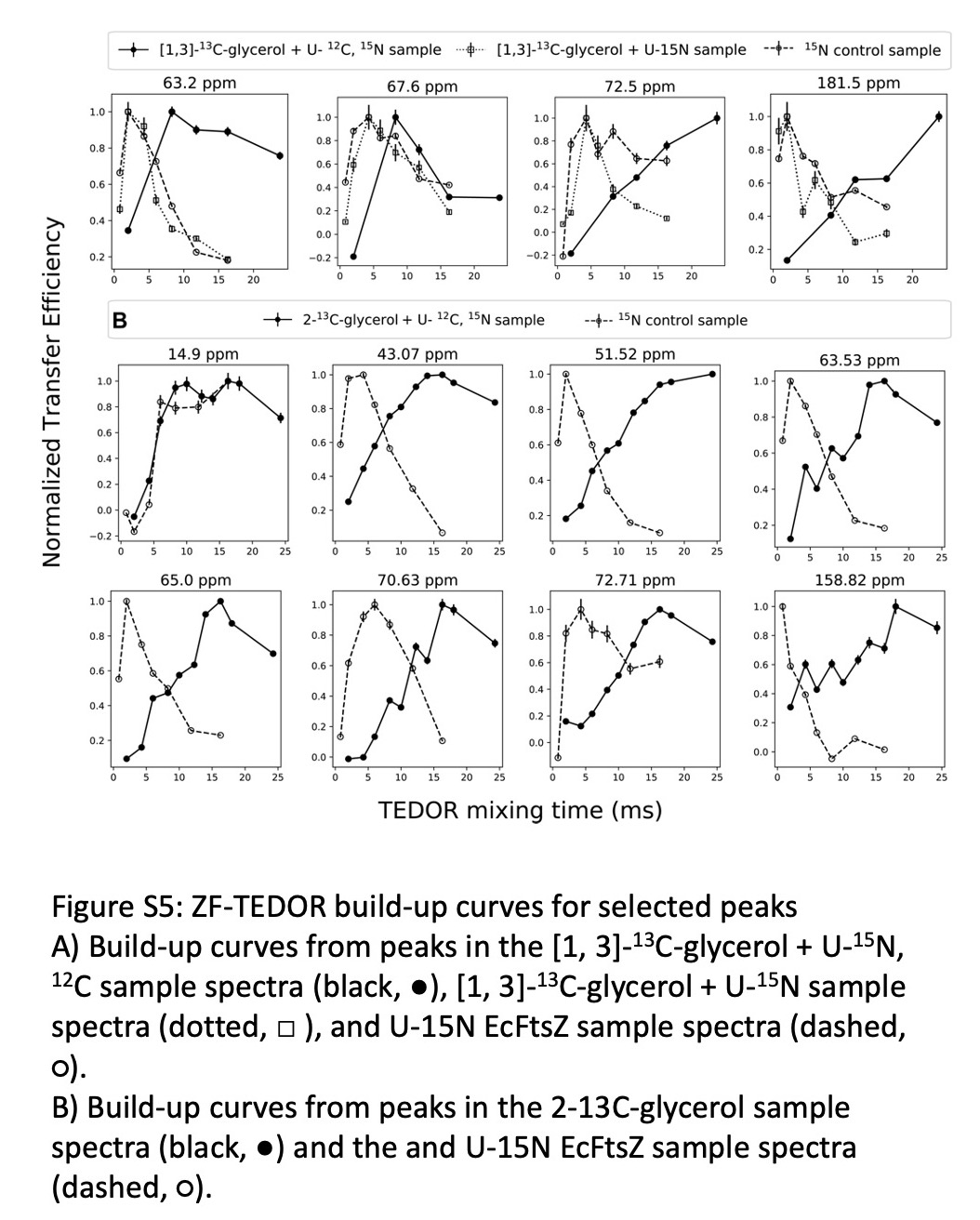

Supplement: FIG S5 [file mbio.02358-22-s0005.jpg]

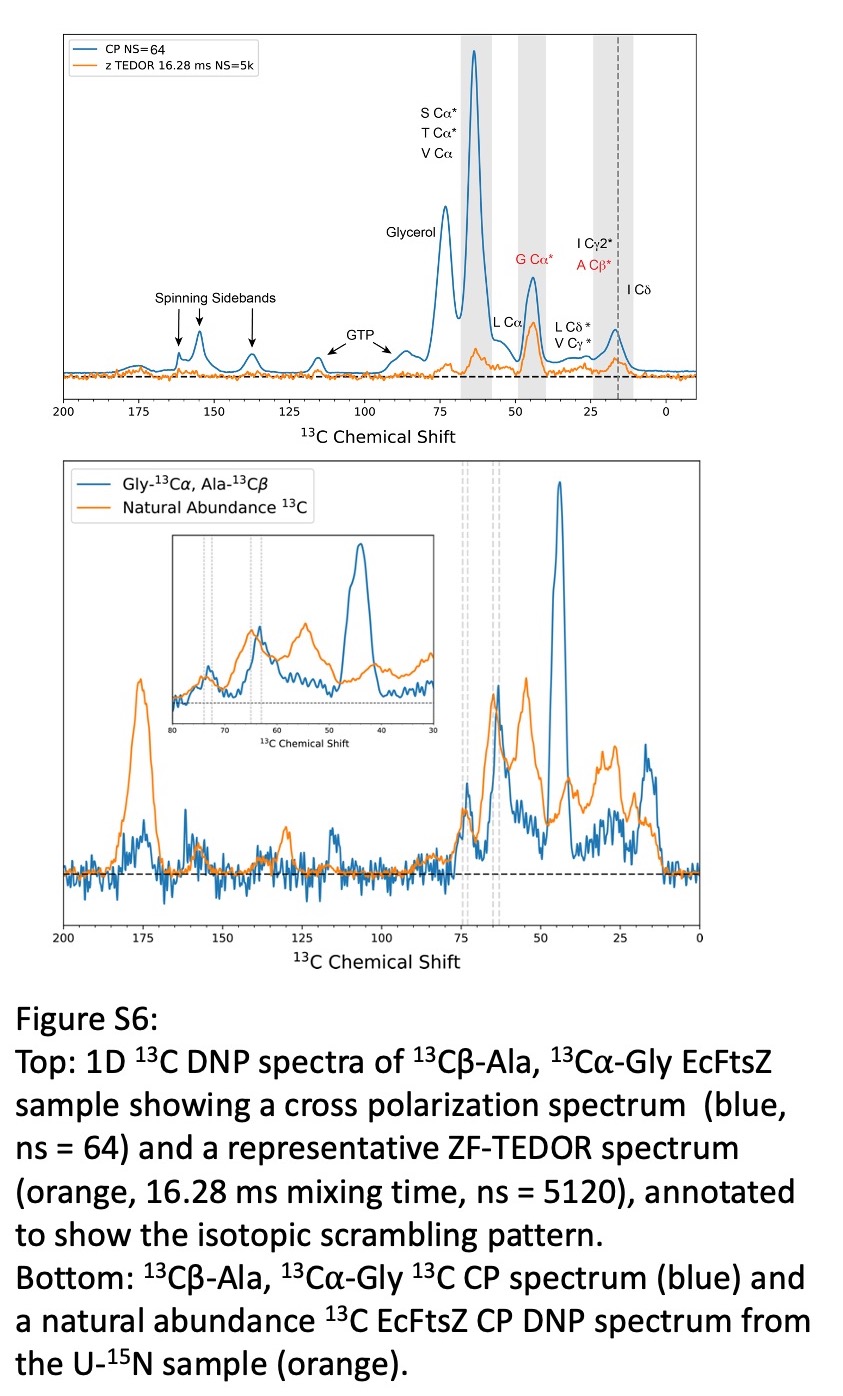

Supplement: FIG S6 [file mbio.02358-22-s0006.jpg]

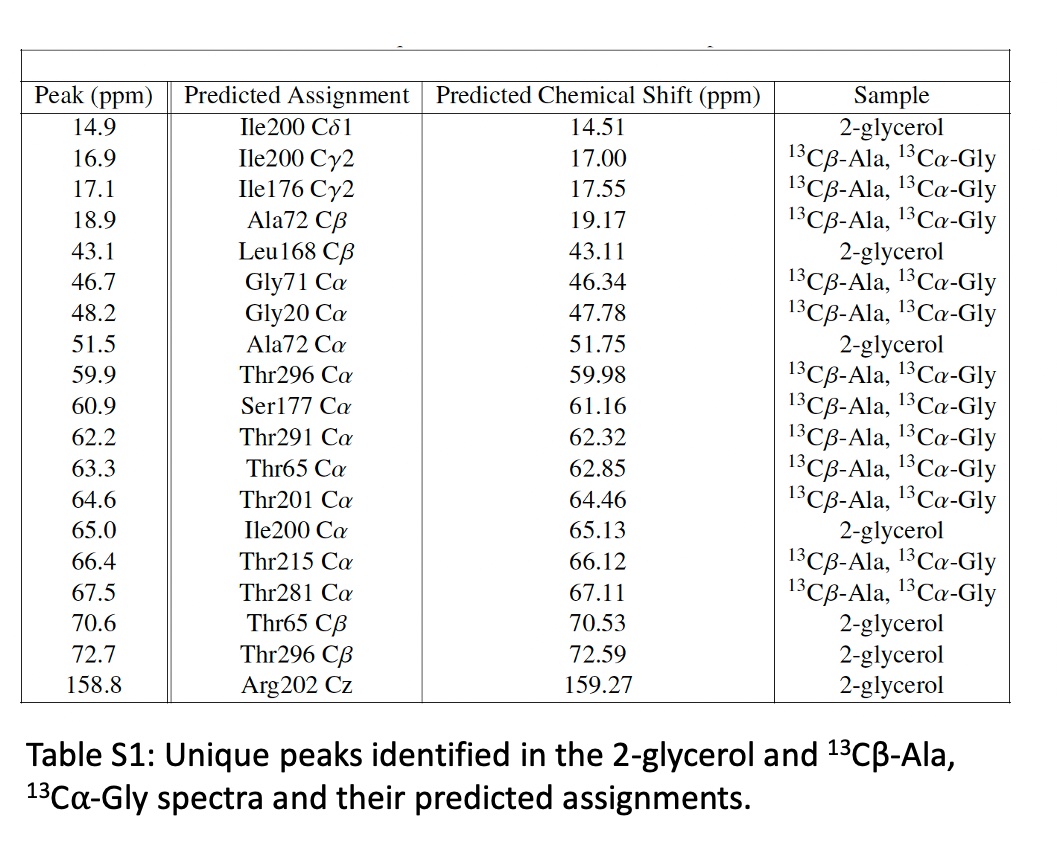

Supplement: TABLE S1 [file mbio.02358-22-s0007.jpg]

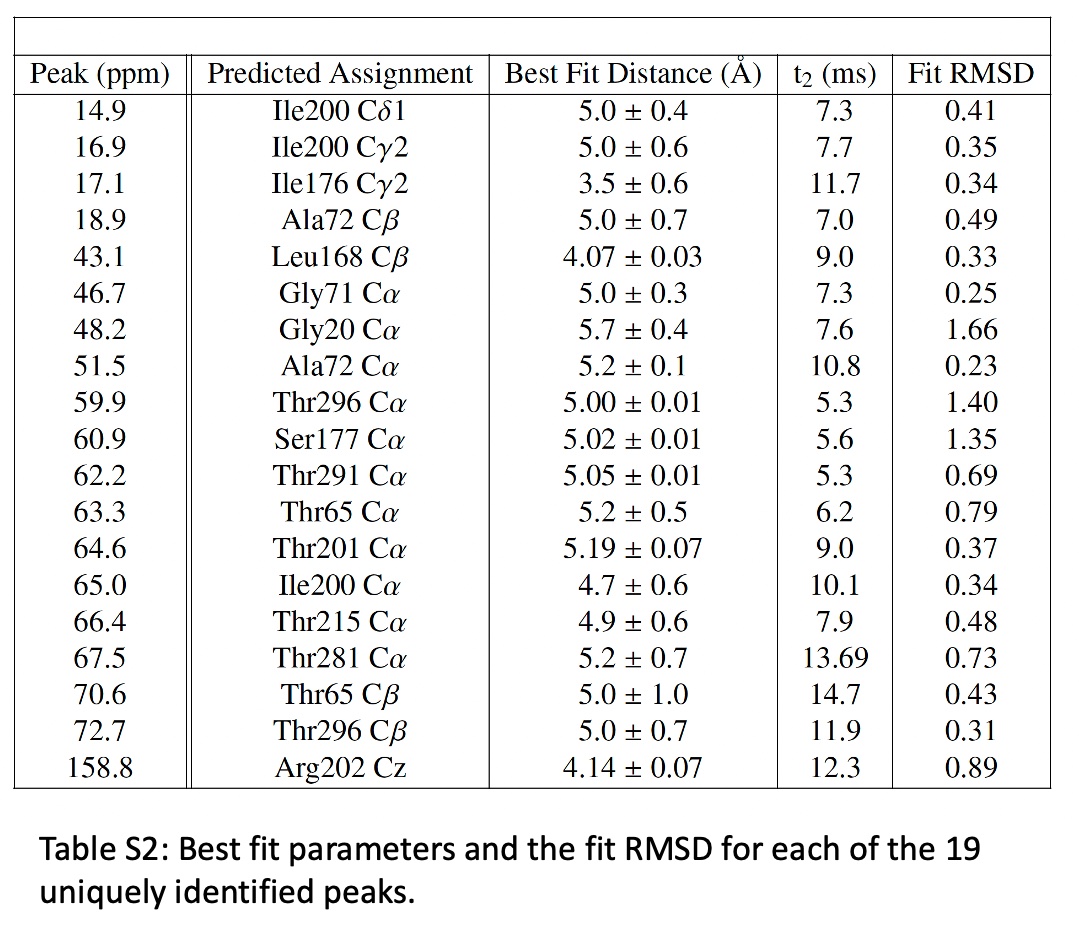

Supplement: TABLE S2 [file mbio.02358-22-s0008.jpg]

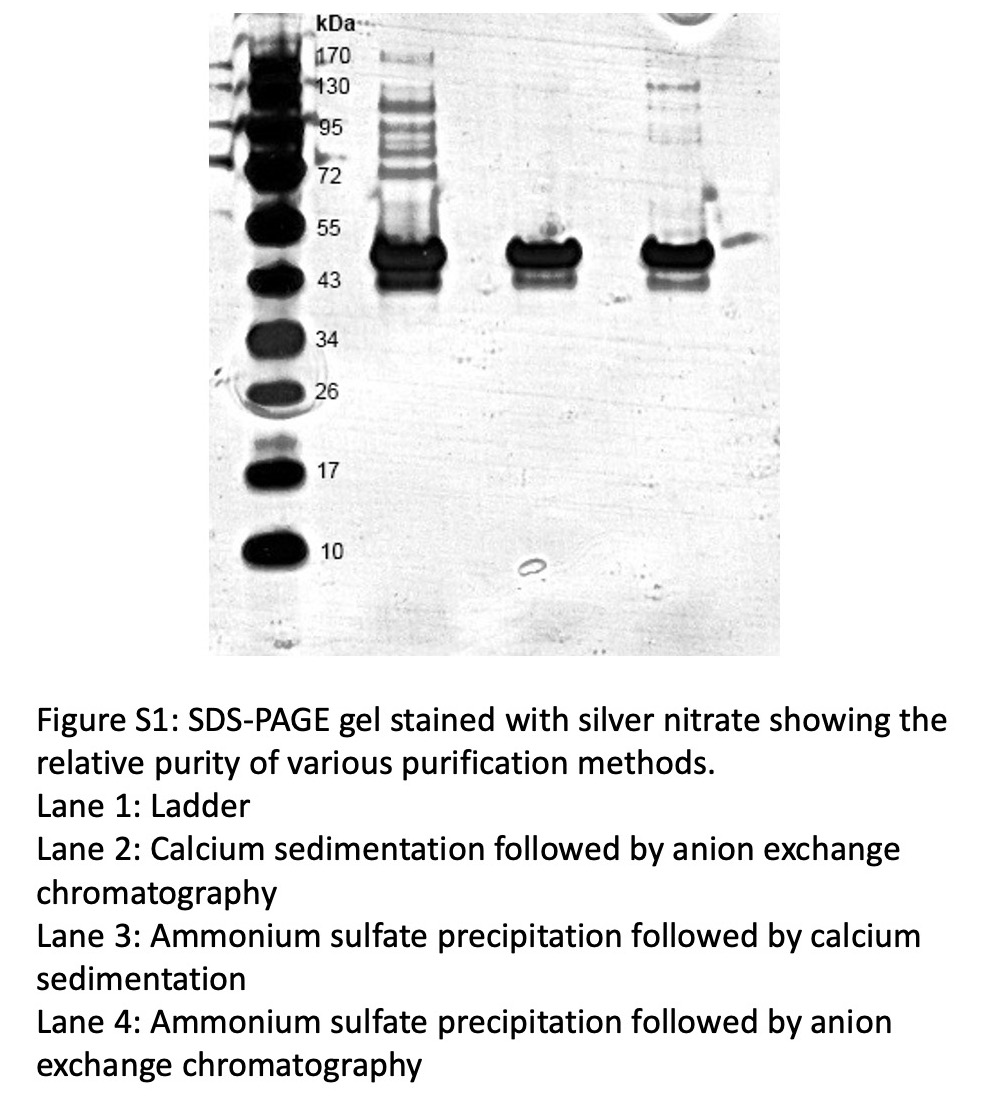

Supplement: FIG S1 [file mbio.02358-22-s0001.jpg]

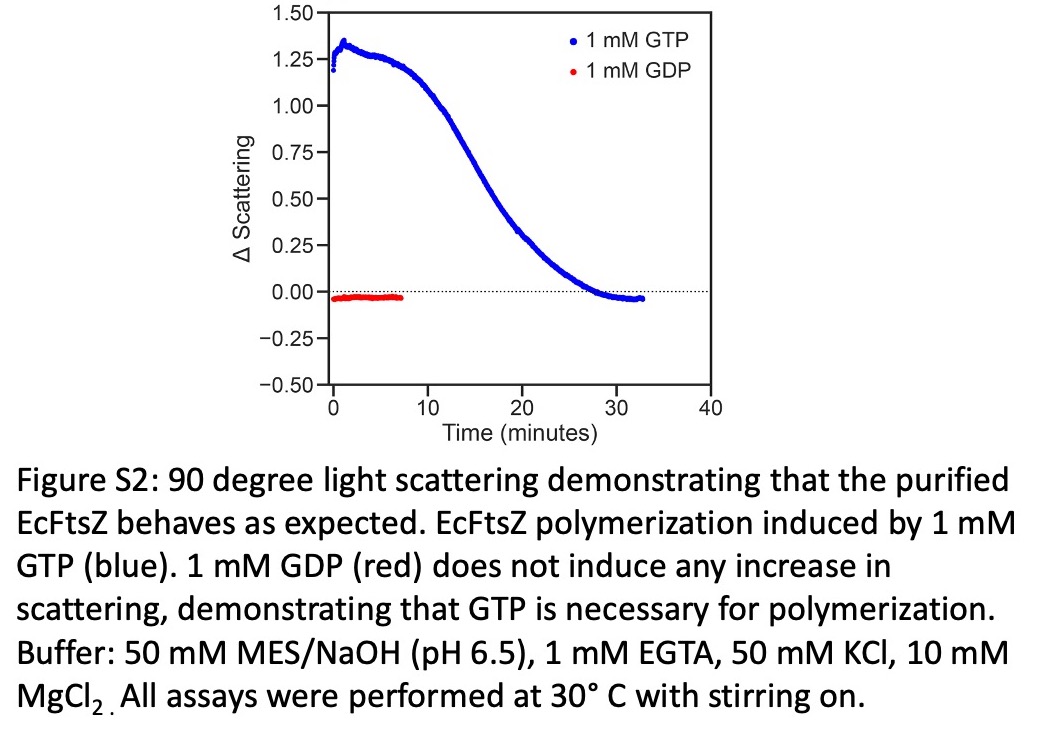

Supplement: FIG S2 [file mbio.02358-22-s0002.jpg]

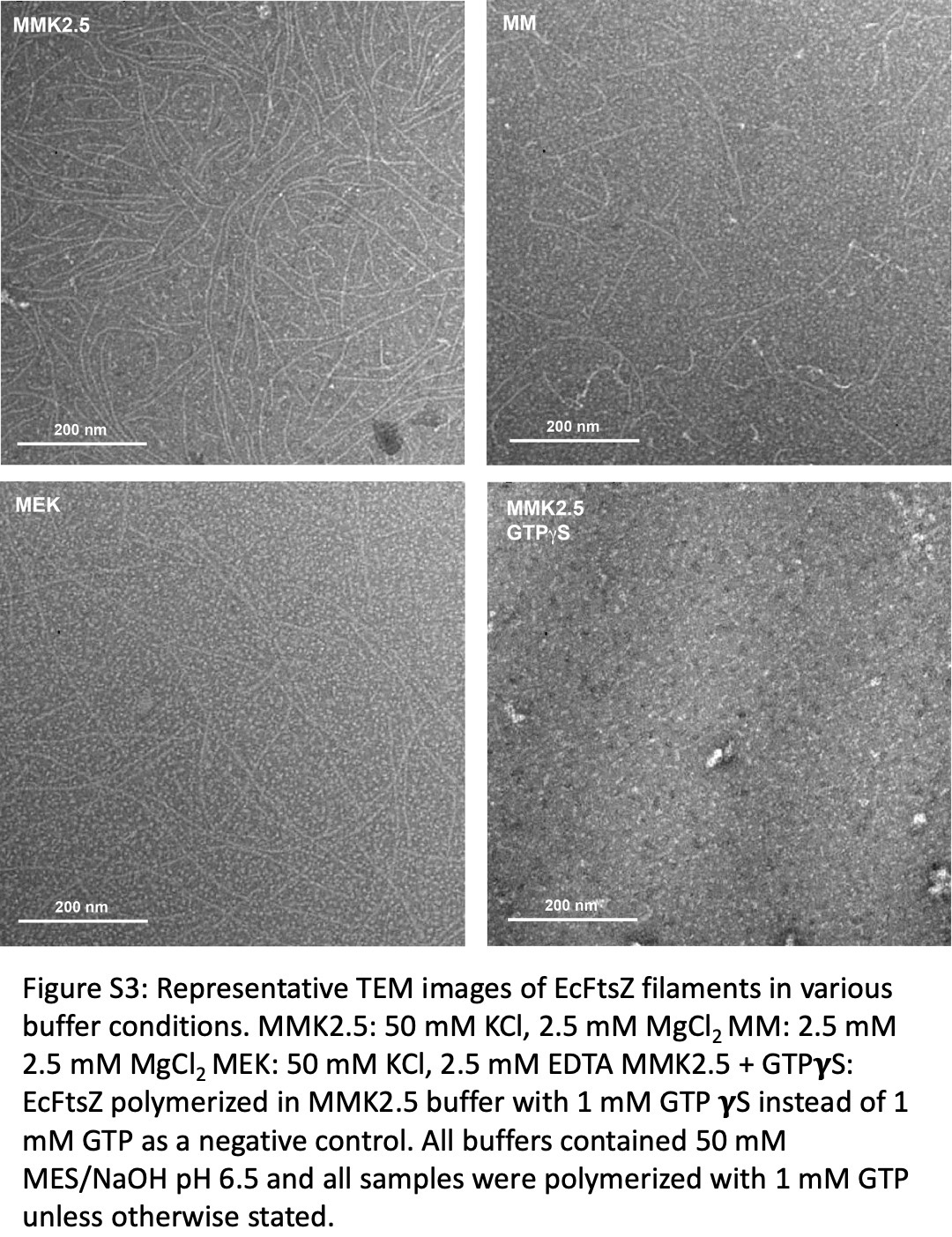

Supplement: FIG S3 [file mbio.02358-22-s0003.jpg]
